# Risk Classification of Food Incidents Using a Risk Evaluation Matrix for Use in Artificial Intelligence-Supported Risk Identification

**DOI:** 10.3390/foods13223675

**Published:** 2024-11-18

**Authors:** Sina Röhrs, Sascha Rohn, Yvonne Pfeifer

**Affiliations:** 1 SGS Germany GmbH, Health and Nutrition, Heidenkampsweg 99, 20097 Hamburg, Germany; sina.roehrs@sgs.com; 2 Department of Food Chemistry and Analysis, Institute of Food Technology and Food Chemistry, Technische Universität Berlin, Gustav Meyer Allee 25, 13355 Berlin, Germany; rohn@tu-berlin.de

**Keywords:** risk assessment, artificial intelligence, food safety, early warning

## Abstract

Foodborne illnesses and mortalities persist as a significant global health issue. The *World Health Organization* estimates that one out of every ten individuals becomes ill following the consumption of contaminated food. However, in the age of digitalization and technological progress, more and more data and data evaluation technologies are available to counteract this problem. A specific challenge in this context is the efficient and beneficial utilization of the continuously increasing volume of data. In pursuit of optimal data utilization, the objective of the present study was to develop a *Multi-Criteria Decision Analysis* (MCDA)-based assessment scheme to be prospectively implemented into an overall artificial intelligence (AI)-supported database for the autonomous risk categorization of food incident reports. Such additional evaluations might help to identify certain novel or emerging risks by allocating a level of risk prioritization. Ideally, such indications are obtained earlier than an official notification, and therefore, this method can be considered preventive, as the risk is already identified. Our results showed that this approach enables the efficient and time-saving preliminary risk categorization of incident reports, allowing for the rapid identification of relevant reports related to predefined subject areas or inquiries that require further examination. The manual test runs demonstrated practicality, enabling the implementation of the evaluation scheme in AI-supported databases for the autonomous assessment of incident reports. Moreover, it has become evident that increasing the amount of information and evaluation criteria provided to AI notably enhances the precision of risk assessments for individual incident notifications. This will remain an ongoing challenge for the utilization and processing of food safety data in the future.

## 1. Introduction

Foodborne diseases persist as a major health and economic challenge. The *World Health Organization* (WHO) estimated that globally, the consumption of unsafe food results in the loss of 33 million healthy life-years annually [1]. One out of every ten individuals becomes ill following the consumption of contaminated food, and USD 110 billion is lost each year in medical expenses and productivity due to unsafe food in low- and middle-income countries [1]. The consequences of consuming contaminated food are various and extensive. They encompass more than 200 diseases caused by chemical or microbiological contaminated food [2]. The spectrum of diseases extends from diarrhea to cancer. Contaminations range from microbial pathogens to heavy metals and can potentially occur at any stage of food production and the supply chain. There are numerous modes of entry into the supply chain. These range from pollution in water, soil, or air to unsafe practices in food processing and storage. However, all these facts underscore the urgent need to enhance food safety worldwide.

The present era of digitalization and technological advancements introduces novel opportunities and methodologies to enhance food safety. The early detection of food risks is becoming increasingly important in the field of food safety. A holistic approach, using horizon scanning, plays a crucial role in this context. In terms of the time perspective, three distinct scenarios can be distinguished: The primary scenario is early warning, which necessitates immediate action. In this situation, the warning signals originate from official sources such as the *Rapid Alert System for Food and Feed* (RASFF) of the European Union. However, in the medium term, the identification of emerging risks is of importance. This comprises risks “resulting from a newly identified hazard to which a significant exposure may occur, or from an unexpected new or increased significant exposure and/or susceptibility to a known hazard”. [3,4]. Screening systems contribute to identifying such risks, enhancing preparedness and facilitating as-early-as-possible risk identification and assessment. The final and most forward-looking aspect pertains to foresight. This domain of horizon scanning is oriented towards the long-term and is crucial for strategic approaches. It deals with driver and scenario analyses for anticipating which food risks may arise in the future. For instance, scientific papers, long-timescale studies, or smaller local events can provide a foundation for the multidisciplinary approaches employed in this context. Drivers are variables that precipitate change. They represent essential factors in the modeling of potential future scenarios. For instance, they include variables like the climate with its temperature alterations, globalization, policy with regard to legal aspects like *Maximum Residue Levels* (MRL) for pesticides or *Maximum Levels* (MLs) for contaminants, and technological progress considering digitalization or innovation [3,5].

Identifying emerging risks is a complex process, which requires the consideration of multiple data and information sources, like legal requirements, incidence notifications, scientific publications and social media news. The volume of available data to enhance food safety is enormous and continuously increasing. This includes data from food safety authorities as well as scientific papers and literature from all kinds of media. For instance, the RASFF only recorded 4361 reports in 2022, compared to 2941 reports in 2016 [6,7].

The increasing amount of available data is a valuable opportunity to detect food risks at an early stage. While the opportunity to enhance food safety is significant, the challenge of effectively utilizing the available data is equally substantial. Therefore, the challenge is to find an effective and time-efficient method for utilizing the data advantageously. One approach would be to implement an AI-supported risk assessment of the data, facilitating the early identification of data with specific relevance through prior categorization. In order to enable this capability, an algorithm must be developed for the risk classification of individual information and incident reports, serving as a basis for AI-driven risk assessment. Different approaches are available for assigning risk categories based on predefined criteria. Here, it is possible to differentiate between qualitative and quantitative tools. Qualitative ones are non-numeric. The generated data are expressed in words and the analysis is interpretive. In contrast, quantitative approaches are more objective. They are numerical, which facilitates a more comparable and reproducible approach. A decision tree would be an example of a qualitative tool. On the other hand, there are quantitative methods for risk categorization, like risk matrices or the use of *Multi-Criteria Decision Analysis* (MCDA) [8]. The latter method enables the most intricate approach among the three options mentioned above. Many factors play a role in the risk assessment of incidence reports. Due to varying degrees of relevance, not all factors are weighted equally in a risk assessment process. This results in the requirement for a quantitative instrument capable of integrating multiple factors with varying weightings. These requirements are met when using MCDA [8,9]. Such evaluations might help to assign certain novel or emerging risks by allocating a level of risk prioritization. Ideally, such indications are obtained earlier than an official notification, and therefore, this method can be considered preventive, as the risk is already identified (e.g., RASFF).

The aim of the present study was to establish a framework for the risk classification of incidence reports, relying on MCDA. The framework should be engineered to enable an AI-supported platform [10] to independently and autonomously perform risk classification of food incident reports in the future. It is hypothesized that an AI-supported application of this framework, utilizing predefined evaluation criteria, will enable the efficient and beneficial use of available data for the risk classification of incident reports. An AI system, into which the newly developed framework can be implemented, is already operating with extensive datasets pertaining to food safety [11].

## 2. Methods

The initial step for an evaluation often involves assessing the probability of the hazard of a (food) product. This represents the probability that a food product is contaminated with a specific hazard, e.g., pieces of metal in salad or chlorpyrifos in sesame seeds. However, this paper deals with the risk assessment of already existing incident reports such as daily RASFF notifications. Therefore, the probability is not considered further, as an event (‘incident’) has already occurred and been published. Thus, this paper will focus on the development of an evaluation scheme, based on MCDA, to classify the risk of incidence notifications. MCDA facilitates the evaluation of multiple criteria with varying weightings by assigning quantitative scores based on the severity of risk associated with each overarching category and its corresponding subcategories. The evaluation scheme should be engineered with such precision that a future AI-supported approach can autonomously analyze and evaluate upcoming incident reports. In such AI systems (e.g., [11]), the datasets encompass a broad spectrum, including legal requirements, scientific publications, and daily incident reports. To facilitate efficient, time-saving, and targeted monitoring of the latter reports, the AI system requires new guidelines, which were developed in the present study as an assessment framework. At this stage, it is essential to recognize that initial data input is required for the effective deployment and functionality of artificial intelligence. To benefit from the use of a future AI implementation, it must first be enabled to operate in a valid way and independently by storing relevant information and work instructions. Initially, prioritization and categorization should be undertaken, which ultimately enable the AI to make informed decisions. The present development of an evaluation scheme serves as a foundation for the intended autonomous assessment of incident reports by AI systems such as the one described by Röhrs et al. [11].

### 2.1. Definition of Overarching Risk Categories, Subcategories, and Weightings

**Figure 1:**
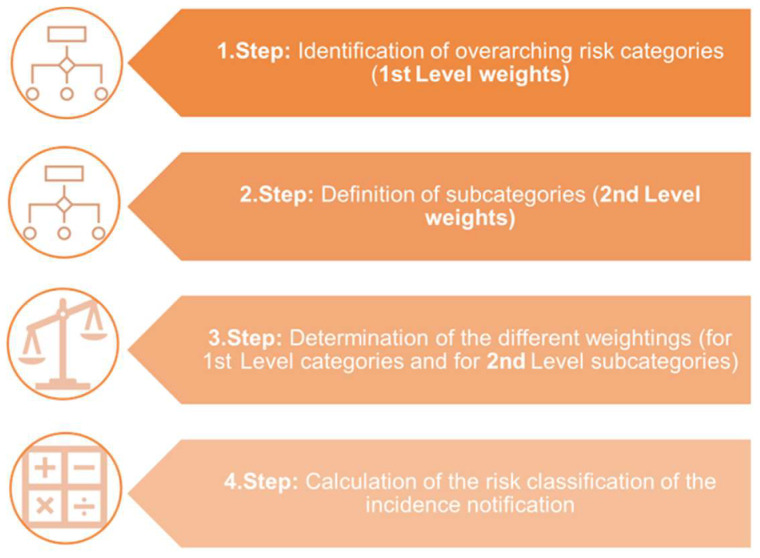
The procedure for the preparation of a risk assessment scheme for incidence notifications.

The intended general process for developing the evaluation scheme is illustrated in Figure 1. The model initiates with the identification/assignment of overarching risk categories. These represent the 1st Level weights for the subsequent assignment and distribution of weightings and the final calculation of the risk of an incident notification. The next step is to define subcategories that are associated with the overarching risk category identified before. These are the 2nd Level weights. The individual overarching risk categories and the associated subcategories are assessed and judged with different evaluation points and weightings based on a certain risk (Step 3, Figure 1). In the subsequent risk calculation, the value of the 1st Level weight is multiplied by the value of the 2nd Level, and the resulting value is incorporated into the total risk calculation of the incident notification (Step 4, Figure 1).

**Figure 2:**
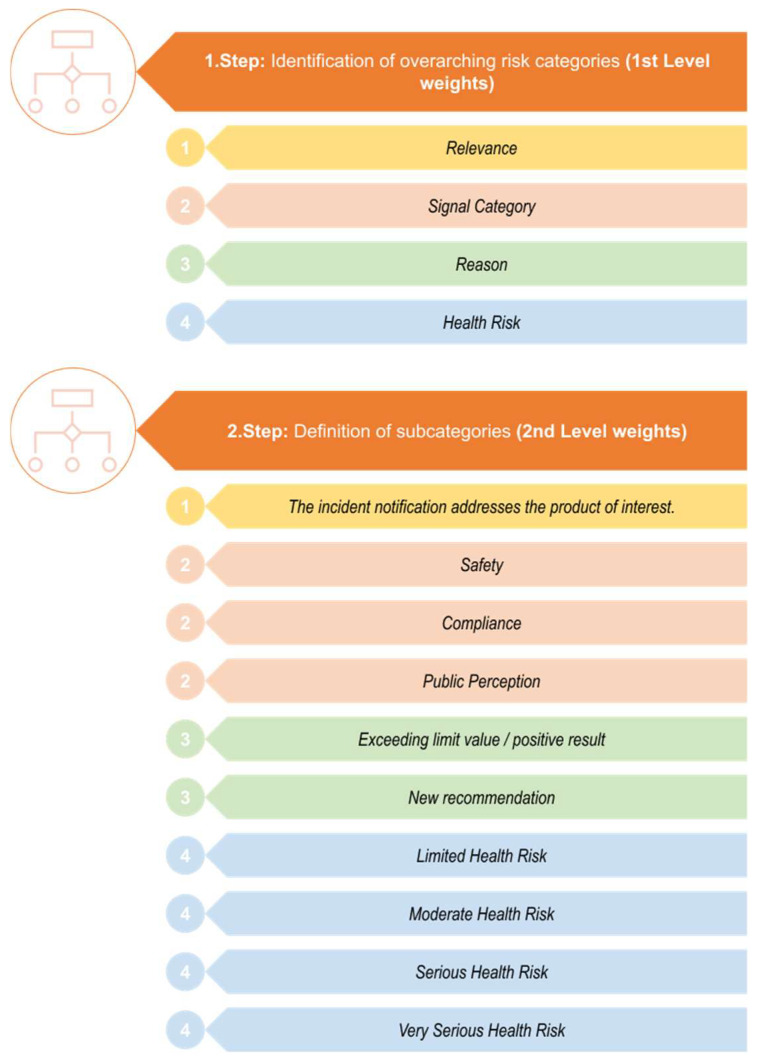
Identification of the overarching risk categories with their subsequently associated subcategories.

In Figure 2, more detailed steps for identifying the overarching risk categories with subsequently associated subcategories are presented.

### 2.2. Specifications of the First Overarching Risk Category and Its Associated Subcategory

**Figure 3:**
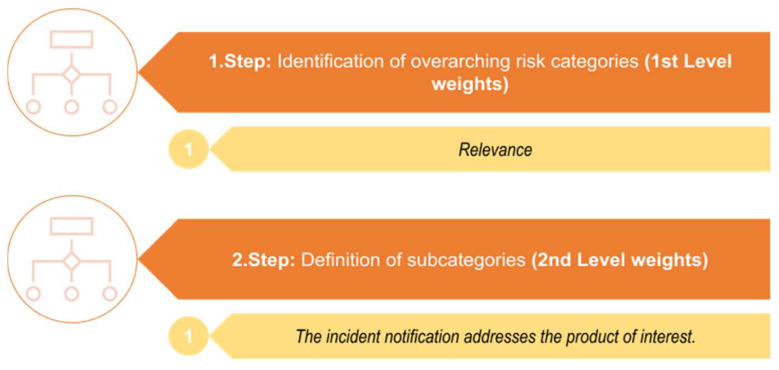
Specification of the overarching risk category *Relevance* and the corresponding subcategory for the risk assessment of incidence reports.

The initial question in the risk assessment of an incidence notification should involve the evaluation of the *Relevance* of the hazard-bearing commodity or product (compare to Figure 1, Steps 1 and 2 and Figure 2, Steps 1 and 2, highlighted in yellow, Figure 3). Risk assessments are usually carried out for specific products or product groups. The associated individual product specification is pivotal, as only reports concerning the products specified are pertinent for the risk assessment. For instance, when the product of interest is sesame seeds, then incidence reports related to sesame seeds are pertinent. Conversely, this means that reports on products outside the previously defined specification are categorized as not so relevant. In the case of sesame seeds, incident reports related, for example, to fruits or vegetables would be excluded because they do not meet the product specifications.

### 2.3. Specifications of the Second Overarching Risk Category and Its Corresponding Subcategories

**Figure 4:**
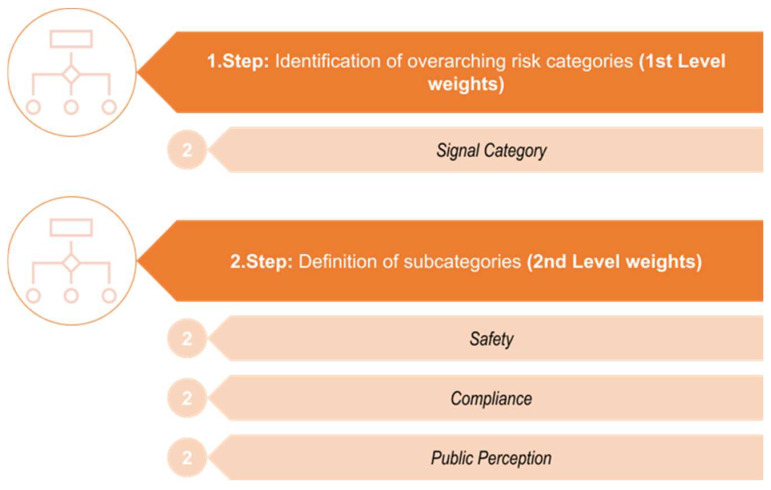
Specification of the second overarching risk category (*Signal Category*) and the corresponding subcategories for the risk assessment of incidence reports.

Once the *Relevance* of the matrix or a certain product or commodity has been identified, an evaluation is carried out within the next overarching risk category, which is the *Signal Category* (compare to Figure 1, Steps 1 and 2 and Figure 2, Steps 1 and 2, highlighted in light orange, Figure 4, upper part). Figure 4 demonstrates the application of Step 1 and Step 2 from Figure 1 for the second overarching criterion, the *Signal Category*, and its associated subcategories. Based on the topic of the incident report, allocation is carried out to one of three subcategories (Figure 4, lower part), named *Safety*, *Compliance*, and *Public Perception*, which are described in more detail below. It is crucial to precisely define the individual overarching categories as well as the corresponding subcategories to enable the AI-algorithms to independently make accurate assignments. Incident reports related to food risks associated with microbiology, allergens, or foreign bodies are categorized under the subcategory *Safety*. Foreign bodies are impurities in food or feed originating from errors occurring during manufacturing, storage, or transportation, e.g., pieces of broken glass, metal, or plastics [12]. Notifications concerning other food risks like pesticide residues, mycotoxins, polycyclic aromatic hydrocarbons, alkaloids, heavy metals, plasticizers, etc., are assigned under the subcategory *Compliance*.

Although both subcategories represent potential hazards in food, it is imperative to adopt a nuanced perspective in this context. This arises from the health risks associated with these subcategories. The risk parameters allocated to the subcategory *Safety* may be more severe, in most cases causing acute health consequences, and no MLs are available compared to those designated to the subcategory *Compliance*. In this subcategory, MRL and ML are considered, and toxicity is, in most cases, a more chronic exposure scenario. The presence of MRL and ML facilitates the classification and risk assessment of hazards related to exposure or consumption. The official European risk assessment of a hazard, as conducted by EFSA experts, along with the derivation of limit values, enables a more precise evaluation of the health risks associated with the consumption of or exposure to a specific quantity of a potentially harmful substance. The three potential hazards (allergens, microbiology, foreign bodies) designated to the *Safety* subcategory do not completely fulfill these requirements. No defined limit values have been established, which is reasonable considering the nature of the three potential hazard groups. For instance, individual variability exists among consumers who are potentially threatened, and informing an individual with a severe peanut allergy that they can safely consume a specific amount of peanut allergen could result in fatal consequences in the worst-case scenario. Similarly, it would be inappropriate to assert that the consumption of a certain amount of *Salmonella* or glass fragments is safe for the public, as the health consequences can be fatal here, too. These circumstances prevent the establishment of the ML, MRL, or Tolerable Daily Intake (TDI) for these three hazard groups, thereby complicating the risk assessment. The potential health risk posed by allergens, foreign bodies, and microbiology is therefore much more difficult to assess and to mitigate. Consequently, a differentiated evaluation of potential hazardous substances through the subcategories *Safety* and *Compliance* is both appropriate and necessary. This consideration is further incorporated into the varying weighting of individual risk parameters during the final calculation of the overall risk (Figure 1, Step 3).

As already mentioned, the assessment of risk arising from an incidence notification should be carried out according to MCDA, a quantitative tool enabling the evaluation of several criteria with different weightings by awarding points for the risk severity of each overarching category and the corresponding subcategories (Figure 1, Step 3). It is important to note that the sum of the individual weightings must add up to 100%. This principle pertains to both the allocation of weightings across overarching risk categories and the distribution within individual subcategories. For better handling, the weighting and subsequent calculation are performed with whole numbers; decimal numbers are not necessary, as only a rough estimation is enough to raise awareness for a more detailed analysis of the risk scenario.

In the evaluation scheme developed, a 100% risk is equivalent to 10 points. Consequently, the sum of the points assigned to the overarching categories must equal 10. The same principle applies to the total point value within a subcategory. For each overarching category, a total of 10 points is allocated among the associated subcategories. The points assigned can be distributed differently depending on the contribution/severity of each subcategory to the overall risk of an overarching risk category.

To emphasize the increased severity of health consequences anticipated within the *Safety* subcategory, a rating of four points is allocated. In contrast, the *Compliance* subcategory is assigned three points, as health consequences within the *Safety* subcategory obviously need a more intense weighting. Here, no MRLs or MLs are available, although the health consequences are often acute and can be life-threatening. The remaining three points are assigned to the third subcategory *Public Perception*. This subcategory exhibits relevance from an economic point of view. Notifications containing the names of companies are assigned to this subcategory. From a corporate perspective, the relevance of the content/topic for a risk assessment of incident reports does not only encompass those addressing limit values and their exceedances in food products. The mention of a company in reports regarding product evaluations conducted by entities such as non-governmental organizations (e.g., Stiftung Warentest or Verbraucherzentrale in Germany, the China Consumers Association, or Consumer Reports in the US) can also result in extensive ramifications. For instance, associating a company with a poor or negative evaluation of one of its products can damage the company’s reputation and negatively impact its sales figures (e.g., consumers might avoid the products of such a company in general). The economic ramifications arising from an inadequate assessment can be extensive, underscoring the necessity of considering these kinds of notifications in the risk assessment of incident reports.

### 2.4. Specifications of the Third Overarching Risk Category and Its Corresponding Subcategories

**Figure 5:**
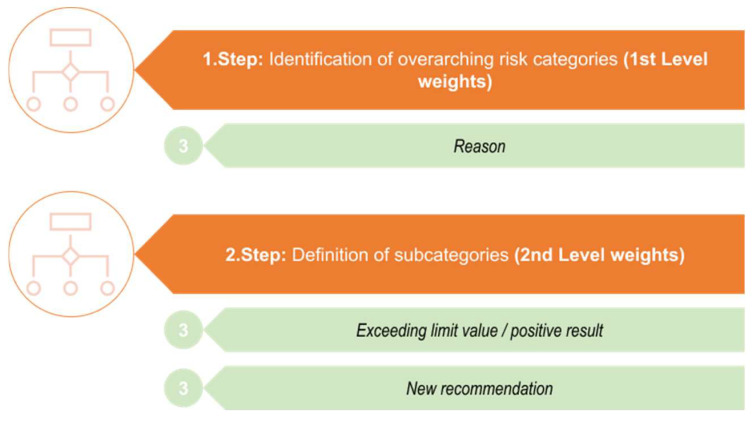
Specification of the overarching risk category *Reason* and the corresponding subcategories for the risk assessment of incidence reports.

The next overarching category for the risk classification of incidence reports is the *Reason* for a report (compare to Figure 1, Steps 1 and 2 and Figure 2, Steps 1 and 2, highlighted in green, Figure 5). Here, it is pertinent to differentiate between two types of reports. One subcategory includes limit value exceedances or positive laboratory findings of risk parameters in food products (Figure 5). The other subcategory comprises reports focusing on new recommendations or limit values for food products (Figure 5).

Varying weightings are applied for the assessment of these two subcategories. It is assumed that exceeding the established limit values presents a higher risk compared to the introduction of new recommendations or future limit values. Hence, the presence of positive laboratory results and the exceedance of limit values are evaluated with six points, whereas new recommendations and future limit values are rated with four points.

### 2.5. Specifications of the Fourth Overarching Risk Category and Its Corresponding Subcategories

**Figure 6:**
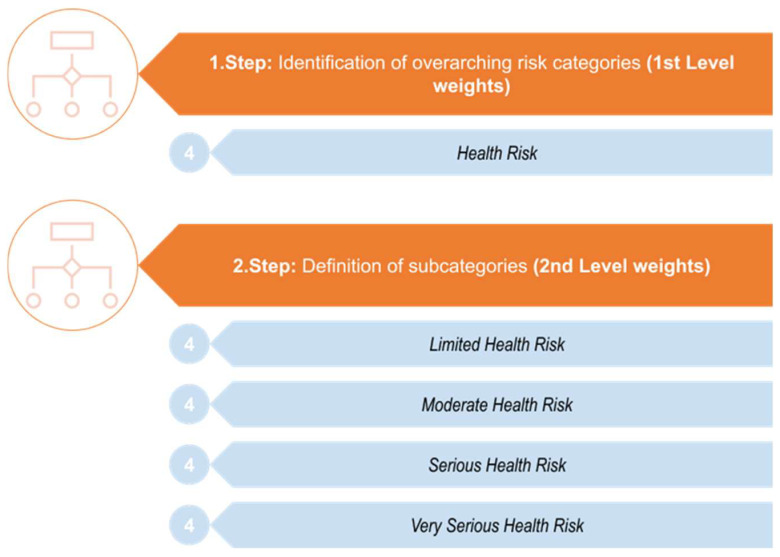
Specification of the overarching risk category *Health Risk* and the corresponding subcategories for the risk assessment of incidence reports.

The final and most important overarching criterion for the risk assessment of an incidence report is *Health Risk* (compare to Figure 1, Steps 1 and 2 and Figure 2, Steps 1 and 2, highlighted in blue, Figure 6). The severity of health risks varies depending on the severity of the topic of an incidence report. For instance, it can be anticipated that the health consequences of food contamination of a microbiological nature (e.g., exceeding cell counts of a pathogenic strain of a bacterium such as enterohemorrhagic *Escherichia coli*) would exceed those resulting from a singular instance of consuming food with elevated but somewhat moderate levels of a pesticide residue. Thus, it is imperative to categorize potential contaminations into distinct risk classifications. This categorization is carried out according to the guidelines of the European Commission [13]. They defined four severity categories for the differentiation of *health risks* arising from a potential hazard. This commission notice from the European Commission encompasses the categorization and comprehensive description of the four severity categories, accompanied by examples of hazards associated with each category. However, this notice does not offer a comprehensive classification of all potential hazards within the four severity categories defined by the European Commission. Nevertheless, the descriptions of the four severity categories issued by the European Commission can be utilized as a framework for the independent assignment of novel or emerging hazards. In the following, the four severity categories will be delineated according to the European Commission’s description. These are then utilized to categorize risks that are not explicitly categorized within the four severity categories defined by the European Commission. The independently derived classifications of unassigned (but potential) hazards are substantiated at relevant points with the corresponding literature, thereby validating the consistency between the classifications and the descriptions of the respective severity categories provided by the European Commission.

The first subcategory is *Limited Health Risk*. Due to the nature of the hazard, there is no problem for the consumer related to food safety, or the hazard can never reach a dangerous concentration or lead to an acute incident. The second subcategory is *Moderate Health Risk*, where no serious injuries and/or symptoms are expected or only when exposed to a very high concentration during a pronounced period of time. However, this description also includes a temporary but clear effect on human health. The third subcategory is represented by *Serious Health Risk* with a clear effect on health with short-term or long-term symptoms, which on rare occasions can result in mortality (e.g., gastroenteritis). Here, the hazard has a long-term effect and/or the maximal dose is not known. The last subcategory is *Very Serious Health Risk*. Within this subcategory, the consumer group is at risk and the hazard can obviously result in acute incidents or even mortality. Moreover, the hazard results in serious symptoms from which mortality may result and/or permanent injuries are possible.

The weightings for the four subcategories are distributed as follows: *Limited Health Risk*—1 point, *Moderate Health Risk*—2 points, *Serious Health Risk*—3 points, and *Very Serious Health Risk*—4 points; keep in mind that the sum has to be 10 points in total.

Based on specifications from the European Commission, individual hazards can be assigned to four subcategories (Table 1).

Some of the potential hazards listed encompass several individual substances and/or necessitate a more detailed description, which can be taken from the following explanations:

*3-MCPD ester and glycidyl ester*: A clear impairment of health with long-term symptoms could be a consequence of exposure [15,16,17]. *Allergens*: For individuals with allergies, the consumption of foods containing allergens can be life-threatening [18]. Examples of allergens are as follows: milk, eggs, fish, crustaceans, mollusks, tree nuts (e.g., almonds, Brazil nuts, cashews, hazelnuts, macadamias, pecans, pistachios walnuts), peanuts, cereals containing gluten (e.g., wheat, rye, barley, oats, spelt), soybeans, sesame seeds, buckwheat, celery, mustard, lupin [19]. With regard to the European food law, fourteen allergens need to be declared within the framework of food labeling regulations [20,21]. *Colorants:* In most cases, food colors are additives used in foods primarily to compensate for color losses due to exposure to light, air, moisture, and temperature variations. They also serve to enhance naturally occurring colors or to add color to foods that would otherwise be colorless or differently colored [22]. Examples of the classification of this hazard are limit value exceedances or the unauthorized use of substances like curcumin (E 100), chlorophyll (E 140), or lutein (E 161b) [23]. *Dioxins*: Long-term exposure to dioxins can lead to serious health effects [24]. This group of approx. 35 toxicologically relevant compounds includes dioxins, dioxin-like PCBs (polychlorinated biphenyls), polychlorinated dibenzo-p-dioxins (PCDDs), polychlorinated dibenzofurans, 2,3,7,8-Tetrachlorodibenzo-p-dioxin (TCDD), and non-dioxin-like PCBs [25]. *Foreign bodies*: These are substances that could cause injury or present a choking hazard, e.g., glass fragments, stones, splinters, thorns, metal, bone fragments, ceramic pieces [26]. *Heavy Metals*: No serious injuries are expected after a single intake of food contaminated with heavy metals, but chronic exposure poses a severe risk. Some of the relevant heavy metals are as follows: arsenic, cadmium, mercury, methylmercury, lead, nickel, and tin [27]. *Large-sized foreign materials/paper*: According to the U.S. Food and Drug Administration, hazardous objects are specified as hard or sharp foreign objects with a maximum size of 25 mm. [28]. Paper does not exhibit these physical properties and can, therefore, be classified under *Limited Health Risk*. Given that this description specifies the maximum size at which a foreign body is considered hazardous, it can be inferred that foreign bodies exceeding this size can be classified under *Limited Health Risk*. *Mycotoxins*: Aflatoxins B1, B2, G1, G2, and M1, ochratoxin A, patulin, citrinin, deoxynivalenol, zearalenone, fumonisins, ergot alkaloids, and alternaria toxins are considered to fall within this group and clearly affect health, causing long-term diseases like cancer [29,30,31]. Here, chronic intoxications are more prominent, but acute risks should not be neglected. *Nitrosamines*: N-Nitrosamines are known for their carcinogenic properties, meaning they have the potential to cause cancer in living tissue, and they encompass a group of several substances (e.g., *N*-nitrosodimethylamine, *N*-nitrosomethylethylamine, *N*-nitrosodiethylamine, *N*-nitrosodipropylamine, *N*-nitrosodibutylamine, *N*-nitrosomorpholine, *N*-nitrosopyrrolidine, *N*-nitrosopiperidine, *N*-nitrosomethylaniline, *N*-nitrososarcosine) [32,33]. *Pathogenic microorganisms*: The consumption of pathogenic microorganisms can result in acute, severe symptoms. Examples of these pathogens are countless with the following being very prominent: *Salmonella*, *Listeria, Clostridia, Escherichia coli*, bacilli, yeasts, molds. *Pesticides*: A comprehensive overview of each of these substances can be found in the EU Pesticides Database [34]. *Polycyclic aromatic hydrocarbons*: These compounds often occur in thermal food processing procedures. The most prominent example is benzo(a)pyrene, exposure to which can lead to serious long-term health effects, mainly diverse forms of cancers [35,36]. *Pyrrolizidine alkaloids* (PAs): The consumption of PAs often results from non-comprehensively cleaned plant raw materials with weeds [37,38]. Twenty-one substances of this group are regulated by the European Contaminants Regulation with an ML [14].

Finally, the weighting and distribution of points for the overarching risk categories has to be carried out. It is important to mention that both the cumulative weightings of individual factors within the subcategories and the aggregated weightings of the overarching criteria must equate to 10. As indicated before, the overarching risk category *Health Risk* has the most substantial impact on determining the overall risk of an incidence report. The assessment reflects the *Health Risk* posed by the hazard. Therefore, the overarching criterion for severity and, thereby, for potential *Health Risk* will be assessed with six points. The remaining four points are allocated a rating of two points for the *Reason* for the notification, and one point each for the matrix’s *Relevance* and the *Signal Category*. This yields the subsequent framework for the risk evaluation of an incident report (Table 2).

### 2.6. Calculation of the Risk Classification (Figure 1, Step 4)

To calculate the risk, the numerical value of the overarching category (1st Level weight) is multiplied by the numerical value of the corresponding subcategory (2nd Level weight). Ultimately, the total risk arising from the incidence notification is determined by summing the four mathematical products computed herein.

Risk categorization = R_1_ × P_1_ + R_2_ × P_2_ + R_3_ × P_3_ + R_4_ × P_4_

where R_1_ to R_4_ are the overarching risk categories and P_1_ to P_4_ are the associated subcategories, while the colored backgrounds in the equation correspond to the four designated areas presented in Table 2. As an example, this means that R_1_ is the point value allocated to the 1st Level weight for the Relevance of the matrix, which is 1 point. P_1_ is the multiplier for R_1_ and is represented by the point value of the 2nd Level weight (the incident notification addresses the product of interest), here, 10 points.

As there are no alternative selection options for all variables, certain point values are treated as fixed constants in the calculation. The variables without selection options consist of the 1st Level weights and the assessment of the *Relevance* of a matrix, as the latter one is the crucial question in the beginning of the assessment of an incidence notification, which was already described in Section 2.2. Given these conditions, the following variables can already be substituted with fixed-point values in the equation.

Risk categorization = 1 × 10 + 1 × P_2_ + 2 × P_3_ + 6 × P_4_

For the 2nd Level weights and thereby for the subcategories of the overarching risk categories Signal Category, Reason for Notification, and Health Risk, more than one option is available for selection. Therefore, these three positions must remain as variables in the equation and can only be replaced by corresponding point values after the evaluation is complete.

The final result determines the risk classification, which is separated into four risk categories (Table 3).

## 3. Results

Before the implementation of the proposed evaluation scheme in an AI-supported database (e.g., [11]) can proceed, its feasibility must be tested manually. In order to test the evaluation scheme presented, it will be applied to different example incidence reports. As previously mentioned, incidence reports and further indications of potential upcoming risks are routinely integrated into AI-supported databases on a daily basis. Hence, the prerequisites for the accessibility and availability of the incidence reports to be evaluated by the AI are already established [11]. The proof-of-concept of the evaluation scheme involves example incident reports concerning sesame seeds [39]. These examples were selected due to their frequent association with various hazards (e.g., pesticides, mycotoxins, or *Salmonella*), providing different examples of applying the framework within the same product group [40,41,42].

### 3.1. Example I

The first incident report to be assessed addresses a potential cadmium Maximum Level exceedance in sesame seeds, as occurred in the year 2023 (notification number 2023.0287) [43]. Based on this incident report, the assessment is conducted according to the framework described in Table 4. The red boxes denote the selected appropriate criteria.

The calculation leads to the following:

Risk categorization = R_1_ × P_1_ + R_2_ × P_2_ + R_3_ × P_3_ + R_4_ × P_4_.

Risk categorization = 1 × 10 + 1 × 3 + 2 × 6 + 6 × 2.

Risk categorization = 37.

This would result in a classification within the low-risk category.

### 3.2. Example II

The second example deals with exceeded MRLs of the pesticides chlorpyrifos and chlorate in sesame seeds, as reported in the year 2024 (notification number 2024.2281) [44].

The assessment of the incidence report using the framework developed results in a risk of 43 points. Compared to the aforementioned example, the *Health Risk* was classified in the *Serious Health Risk* subcategory. For instance, the consumption of chlorpyrifos-contaminated sesame seeds could induce a clear effect on health, like cramps, diarrhea, or blurry vison [45]. In this example, the overall result leads to a classification within the Moderate Health Risk category.

### 3.3. Example III

In July 2024, *Salmonella* was detected in sesame seeds from Sudan (notification number 2024.5236) [46].

This results in a calculated risk of 50 points for the incidence report and a classification within the high-risk category. In comparison to the two previous examples, *Health Risk* in this case was classified as Very Serious, as the consumption of *Salmonella*-contaminated food can result in very serious health consequences, like typhoid fever, which can be life-threatening [47].

## 4. Discussion

The primary objective of this study was to develop a risk assessment scheme for food incident reports that has sufficient precision for later implementation into an AI system capable of autonomously applying this approach as a new feature. The potential target AI-supported platform, Digicomply, which has been previously described in detail by Röhrs et al. (2025) [11], already exists. Given the continually growing volume of food safety data, such as daily incident reports, assessing emerging risks to identify and prioritize high-risk incidents promptly, allowing for rapid responses and the implementation of countermeasures, is a time-intensive task. This is where the role of AI becomes crucial. Although the development of the risk assessment scheme presented herein is not obviously linked directly to the final comprehensive AI application, the present assessment approach is also intended to operate in an automated manner and to be integrated into the overall algorithm of the overarching future system. Automated risk assessment optimizes time efficiency and facilitates the earlier detection of potentially high-risk incidents, thereby enabling the more rapid deployment of countermeasures when required. To make this feasible, the present assessment scheme must be precisely defined and follow clear operational guidelines; otherwise, AI execution will not be possible. It is essential to understand that, for holistic AI to function effectively, it must first be provided with extensive information. The overall AI platform must be programmed with clear instructions on how to respond in various situations. To assess whether the information and work instructions are sufficiently precise and comprehensive, the risk assessment scheme, intended for future implementation in an AI-supported database, was initially tested manually. The proof-of-concept for the developed framework using different incidence reports on sesame seeds exhibited promising results in the calculation and classification of the overall risk for each individual notification.

Based on the calculations, all three example incidence reports could be categorized into an appropriate risk category. However, it should be noted that the developed framework is relatively general and, in some cases, like in the evaluation of the *Public Perception* subcategory, lacks specificity, as described below.

The implementation of the framework for AI becomes increasingly complex as the individual evaluation criteria become more specific and diverse. Firstly, the individual evaluation criteria must be precisely defined as distinct overarching categories and corresponding subcategories to enable the AI to operate within a clear and unambiguous framework. For this purpose, it is essential to provide the AI with as much information as possible. As machine learning algorithms learn through data and constant learning, the evaluation of hazards/commodities will become more and more precise with proceeding time. However, for a more precise and comprehensive assessment, additional information, like company-specific data, would be necessary and helpful. For instance, this becomes particularly clear in the assessment of the *Public Perception* subcategory. In the framework presented here, the sole criterion for a classification within this subcategory is the specific company name. However, a more comprehensive set of information would be required to accurately assess the potential economic risk arising from the incidence report. For a thorough assessment, factors such as the reliability and reach of the publishing source should be considered. For instance, when the source is a dubious publisher with a small readership, the potential economic damage can be assumed to be low. Conversely, when the source is a recognized non-governmental organization (e.g., in Germany, *Stiftung Warentest* or *Verbraucherzentrale;* in the *US, Consumer Reports*; the *China Consumers Association* in China; *Organización de Consumidores y Usuarios* in Spain; or *Que Choisir* in France), the potential economic impact is likely to be higher. Moreover, the company’s sales figures in the relevant market as well as the market share are relevant aspects. When the report addresses a market, where a company has a substantial market share and high sales figures, negative reports like incidence notifications can result in more serious damage to a company’s sales and reputation compared to a market with only a marginal presence. In the latter case, conducting an assessment proves challenging as it relies on the company’s willingness to disclose data that may not be publicly accessible. In addition to data provision, specific company criteria must be defined for the assessment, considering that small and large companies may have varying perspectives on what constitutes high sales figures or a high market share. In summary, achieving the most accurate assessment possible is a highly complex endeavor that necessitates abundant information and precise definitions.

Moreover, despite the quantitative evaluation scheme, the framework presented is susceptible to a certain degree of subjectivity. For instance, this becomes evident in the categorization of individual hazards into the four subcategories for Health Risk. As only certain hazards have been categorized into the four risk categories by the European Commission, unallocated risks, like allergens, were categorized according to the descriptions provided for these categories (c.f., Table 1) [13]. While it is evident that allergens, such as those capable of inducing an anaphylactic shock and potentially leading to mortality (e.g., peanut allergy has a higher risk than buckwheat allergy), pose a very serious risk, the classification of heavy metals is debatable (mercury and cadmium toxicity have a far smaller severity than peanut allergy).

The group of heavy metals encompasses several substances with diverse toxicological evaluations and ML. For example, the element cadmium, according to the *International Agency for Research on Cancer*, is classified as carcinogenic to humans, and the element lead as possibly carcinogenic, while the element mercury is non-carcinogenic [48]. Generally, the assessment of CMR (carcinogenic, mutagenic, and reprotoxic) substances is difficult, as exposure doses cannot be assessed easily and concentrations leading to severe outcomes (i.p. cancer) are dependent on many individual factors. This implies that assessment models need to be more conservative and set under continuous monitoring processes.

Obviously, ML and MRL also vary between different substances. For instance, in the European Union, the ML for mercury in salt is 0.1 mg/kg, for arsenic it is 0.5 mg/kg, and it is between 1 and 2 mg/kg for lead [14]. Moreover, there are also discrepancies between different markets. For instance, the ML for lead in salt in India is 2 mg/kg. Consequently, the assessment of heavy metals is highly complex, as here, they are considered a group and not single substances. For instance, high concentrations of arsenic, often found in drinking water, can induce acute toxicity with symptoms like vomiting and diarrhea and possibly death [49,50]. In contrast, the intake of cadmium, for example, through contaminated chocolate, poses a risk for chronic toxicity due to long-term exposure, which can lead to outcomes such as cancer [51,52]. However, the described exposure consequences represent worst-case scenarios, and according to the European Commission’s definition of a Moderate Health Risk, classification within this risk category seems appropriate, as serious injuries and/or symptoms are possible but unlikely from a single exposure to these chemical elements.

Even at a macromolecular level, clearly defining individual hazards and categorizing them into the four *Health Risk* subcategories (c.f., Table 1) can be challenging. For instance, a critical point for the AI-supported implementation of the framework is the assessment of foreign bodies. The term “foreign bodies” is broad and encompasses a wide range of potential hazards. These range from a low health risk to life-threatening conditions, depending on the nature of the foreign body. Therefore, it is crucial to precisely specify the various assessments of foreign bodies to ensure appropriate classification by AI. Paper, soft plastic, and large-sized foreign materials are classified as Low Health Risk by the European Commission [13]. This classification is justified, as it can be assumed that either the nature of the foreign body presents no health risk upon ingestion, or the particles are sufficiently large to be visible and can be removed prior to consumption. However, it is also essential to clearly define what constitutes “large-sized foreign materials”. Even with a clear definition, it remains uncertain whether the source of the incident notification provides sufficient information for the AI to make a comparison that would enable accurate classification into the appropriate *Health Risk* subgroup. Such a situation also applies to the assessment of other hazards. For instance, sometimes, the incident source does not provide the type of bacteria, like *Salmonella* or *E. coli*. It only gives the generic term ‘bacterial contamination’. At this point, it becomes evident that even with extensive preparatory actions taken for the AI, accurate classification can still be challenging in certain situations, and a human assessment may be required. Despite the availability of comprehensive data, the notion of AI functioning entirely autonomously without human intervention remains difficult. Moreover, ensuring precise risk assessment will remain a challenge due to the ongoing demands of data provision and processing for food safety in the future. For the most accurate risk assessment of incident notifications by AI, it remains essential to provide comprehensive information and clearly defined evaluation criteria.

Future considerations should include expanding the scope of the risk assessment to encompass additional areas and integrating as much relevant data as possible. In addition to the risk assessment of an incident notification provided, other factors could be considered, such as the timing of non-conformity detection. Whether the “risky” goods are still on board the vessel or have already entered circulation is highly relevant. Moreover, incorporating audit data could provide valuable insights. The degree of compliance with regulations by food producers, including hygiene standards, plays a critical role in ensuring food safety. The volume of available data that can contribute to food safety is continuously increasing. Without the support of AI, processing all of these data would be highly time-consuming, with the identification of critical signals or correlations occurring too late, potentially delaying the implementation of necessary countermeasures. With the assistance of AI, there is a realistic opportunity to consider and leverage this large volume of data for effective risk assessment. As demonstrated by the development of the risk assessment scheme in the present study, initially, it is time-consuming to organize and store all information and work instructions in a format that can be utilized by the AI system. However, the subsequent time savings and improved responsiveness to critical food incidents are indisputable. Even with prior familiarity with the assessment scheme presented here, conducting a manual risk assessment of an incident report requires approximately 5 min. The incident report must first be thoroughly reviewed and comprehended to enable an accurate and appropriate assessment across all criteria of the evaluation scheme. Finally, the overall risk must be calculated to determine the appropriate risk categorization. Given the aforementioned steps involved, a 5 min timeframe is relatively brief for completion. It can therefore be assumed that an individual lacking extensive familiarity with the assessment scheme may require additional time to accurately assess the risk of a single incident report. For example, the RASFF recorded 29 notifications on 8 November 2024, 18 notifications on 7 November 2024, and 19 notifications on 6 November 2024. The average number of incident reports over this three-day period is thus calculated to be 22 reports per day. Assuming that an individual familiar with the assessment scheme requires approximately 5 min to evaluate the risk of an incident report, an average of 110 min would be needed to assess approximately 22 incident reports per day. Rather than allocating these 110 min to the manual risk assessment of incident reports, this time could be more effectively utilized to further examine reports classified as moderate or high risk by the AI and, if necessary, initiate appropriate countermeasures. Therefore, the AI-based risk assessment of incident reports not only saves time, but also facilitates the earlier implementation of countermeasures, thereby improving food safety.

However, as previously mentioned, the evaluation scheme developed here should be considered a starting point. A wealth of additional data are available that can be utilized for risk assessment and, ultimately, to enhance food safety. The inclusion of more data leads to a more precise risk assessment, and with the support of AI, it is now feasible to incorporate these data into the evaluation process. Thus, the next step is to integrate additional information and work instructions into the AI system to enable the incorporation of further data, such as audit data, into the assessment process.

## 5. Conclusions

Utilizing an AI-supported algorithm for the assessment of food safety incident notifications is an efficient and time-saving approach for preliminary risk categorization of the reports. It facilitates the rapid identification of relevant notifications related to predefined subject areas and thereby detects notifications that necessitate further examination. However, it is important to note that the AI requires comprehensive information and detailed evaluation criteria to maximize the accuracy of its risk assessments and classifications. The provision and processing of the required data will remain a challenge in the future. It should also be considered that, despite the availability of extensive information for the AI, there may still be situations where human intervention remains necessary for an appropriate risk classification.

The analyzed example incident reports could be easily and appropriately categorized by risk using the evaluation scheme developed. Consequently, the practicability of the evaluation scheme was confirmed. Of course, this was carried out manually, as described herein. In the future, this assessment should be also conducted independently by AI. Its implementation in AI-supported databases is deemed both advantageous and feasible (e.g., [11]). Additionally, the initial examples already offer a preliminary indication of the contributions that a future AI-supported evaluation of incident reports can make regarding time savings and efficiency. The application provides immediate clarity on which incident reports pose a serious risk and necessitate further examination, as well as those that are deemed less risky. The incidence report on cadmium in sesame seeds was classified as low risk; thus, it does not necessarily require further investigation. When the AI-based risk assessment indicates that the incidence report has been classified as low risk, a closer examination may not be required. This, in turn, saves time and facilitates the efficient management of the continuously increasing volume of available food safety data. It is important to note that the daily volume of new incident reports is not limited to three reports, as presented is the examples given in this study. The daily volume of incoming incident notifications is substantially higher than the three examples discussed in this analysis, which can ultimately be reflected in the potential time savings. For the single incident report classified as low risk in this context (as shown in Example 1), the time saved from not requiring a closer investigation may not be substantial. However, when aggregated across all incident reports evaluated in a single day, the cumulative time savings will be considerable. It is also essential to note that the autonomous AI-supported assessment enables the more rapid detection of hazardous incidence reports. The more quickly these incident reports are recognized, the more rapidly countermeasures can be taken. The more rapidly this process is carried out, the more effectively individuals can be prevented from consuming contaminated food and being exposed to health risks.

Novel and emerging hazards can be identified as well. Based on the historical context, one cannot recognize if there is a developing risk or if actual data can imply that one needs to take a closer look to the risk assessment. An initial categorization might help to assign a certain risk by allocating a level of risk prioritization. Ideally, such indications are obtained earlier than an official notification, and therefore, this method can be considered preventive, as the risk is already identified (e.g., RASFF).

Overall, the benefits of integrating AI into the risk assessment of incident notifications are convincing, although human involvement may occasionally be valuable. Especially in cases where carcinogenicity or acute toxicity can be an outcome, additional reviewing seems to be indicated.

## Figures and Tables

**Table 1:** The assignment of potential hazards to the four Health Risk subcategories. Hazards already assigned by the European Commission are labeled accordingly. Example hazards not yet explicitly assigned by the European Commission are categorized based on the descriptions of the individual subcategories. The listed hazards encompass substances regulated under the European Contaminants Regulation as well as other potential hazards [14].

*Limited Health Risk*	*Moderate Health Risk*	*Serious Health Risk*	*Very Serious Health Risk*
Paper ^1^	Small pieces ^1^	Pesticide residues ^1^	Allergens
Soft plastic ^1^	Heavy metals	Dioxins ^1^	Pathogenic microorganisms
Colorants ^1^	Erucic acid	Mycotoxins ^1^	Viruses (e.g., norovirus, Hepatitis A virus)
Large-sized foreign materials ^1^	Δ^9^-THC ^3^	MOSH/MOAH ^4^	Marine biotoxins (e.g., tetrodotoxin)
Packaging (corrosion, damaged)	Irradiation	*3-MCPD* ^5^ *ester and glycidyl ester*	Foreign bodies (with direct health danger)
Sensing (smell, taste, color)	Nitrate	Polycyclic aromatic hydrocarbons	
Labeling (except allergen labeling)	Melamins	Alkaloids (e.g., pyrrolizidine alkaloids, tropane alkaloids, opium alkaloids)	
Others (e.g., lack of traceability, incorrect/missing certificates)	Caffeine	Perfluoroalkyl substances ^2^ (e.g., PFOS, PFOA, PFNA, PFHxS)	
	Veterinary drugs (e.g., antibiotics)	Acrylamide	
		Bisphenol A	
		Perchlorate	
		Hydrocyanic acid	
		Nitrosamines	

^1^ Assignment by the European Commission [12]. ^2^ PFOS: perfluorooctane sulfonate; PFOA: perfluorooctanoic acid; PFNA: perfluorononanoic acid; PFHxS: perfluorohexanesulfonic acid. ^3^ Δ^9^-THC^3^: Delta-9-tetrahydrocannabinol. ^4^ MOSH/MOAH: Mineral Oil Saturated Hydrocarbons/Mineral Oil Aromatic Hydrocarbons. ^5^ 3-MCPD: 3-monochloropropane-1,2-diol.

**Table 2:** Framework for the risk evaluation of an incident report.

Criteria	1st Level Weights	2nd Level Weights
***Relevance of matrix***	1	
The incident notification addresses the product of interest.		10
***Signal Category***	1	
*Safety*		4
*Compliance*		3
*Public Perception*		3
***Reason for notification***	2	
Exceeded limit value/positive results		6
New recommendation		4
***Health Risk***	6	
*Limited Health Risk*		1
*Moderate Health Risk*		2
*Serious Health Risk*		3
*Very Serious Health Risk*		4

**Table 3:** Classification of the four risk categories for assessing the risk of an incidence notification.

Negligible Risk	Low Risk	Moderate Risk	High Risk
<31 points	32–37 points	38–43 points	>44 points

**Table 4:** Risk evaluation of the incident report on cadmium in sesame seeds (notification number 2023.0287).

Criteria	1st Level Weights	2nd Level Weights
***Relevance of matrix***	1	
The incident notification addresses the product of interest.		10
***Signal Category***	1	
*Safety*		4
*Compliance*		3
*Public Perception*		3
***Reason for notification***	2	
Exceeded limit value/positive results		6
New recommendation		4
***Health Risk***	6	
*Limited Health Risk*		1
*Moderate Health Risk*		2
*Serious Health Risk*		3
*Very Serious Health Risk*		4

## Data Availability

The original contributions presented in the study are included in the article, further inquiries can be directed to the corresponding author.
